# Crystal structure of (*E*)-*N*-(3,3-di­phenyl­allyl­idene)-9-ethyl-9*H*-carbazol-3-amine

**DOI:** 10.1107/S2056989015005770

**Published:** 2015-03-28

**Authors:** Kannan Thirumurthy, Ganesamoorthy Thirunarayanan, S. Murugavel

**Affiliations:** a Department of Chemistry, Annamalai University, Annamalainagar 608 002, Chidambaram, Tamilnadu, India; b Department of Physics, Thanthai Periyar Government Institute of Technology, Vellore 632 002, India

**Keywords:** crystal structure, carbazole, 9-ethyl-9*H*-carbazol-3-amine, C—H⋯π inter­actions

## Abstract

In the title compound, C_29_H_24_N_2_, the C=N bond of the central imine group adopts an *E* conformation. The dihedral angles between the mean plane of the essentially planar carbazole ring system [r.m.s. deviation = 0.039 (2) Å] and the two phenyl rings of the 3,3-di­phenyl­allyl­idene unit are 75.9 (1) and 64.6 (1)°. In the crystal, mol­ecules are linked by C—H⋯π inter­actions, forming a three-dimensional supra­molecular network.

## Abstract 2

In the title compound, the carbazole ring system is essentially planar (maximum deviation = 0.025 Å). The crystal packing is stabilized by inter­molecular C—H⋯π inter­actions, forming a three-dimensional supra­molecular network.

## Chemical context   

Carbazole and its derivatives have become quite attractive compounds owing to their applications in pharmacy and mol­ecular electronics. It has been reported that carbazole derivatives possess various biological activities, such as anti­tumor (Itoigawa *et al.*, 2000 ▸), anti-oxidative (Tachibana *et al.*, 2001 ▸), anti-inflammatory and anti­mutagenic (Ramsewak *et al.*, 1999 ▸). Carbazole derivatives also exhibit electroactivity and luminescence properties and are considered to be potential candidates for electronic devices such as colour displays, organic semiconductor lasers and solar cells (Friend *et al.*, 1999 ▸). These compounds are thermally and photochemically stable, which makes them useful materials for technological applications. For instance, the carbazole ring is easily funtion­alized and covalently linked to other mol­ecules (Díaz *et al.*, 2002 ▸). This enables its use as a convenient building block for the design and synthesis of mol­ecular glasses, which are widely studied as components of electroactive and photoactive materials (Zhang *et al.*, 2004 ▸). Against this background, and in order to obtain detailed information on mol­ecular conformations in the solid state, X-ray studies of the title compound have been carried out.
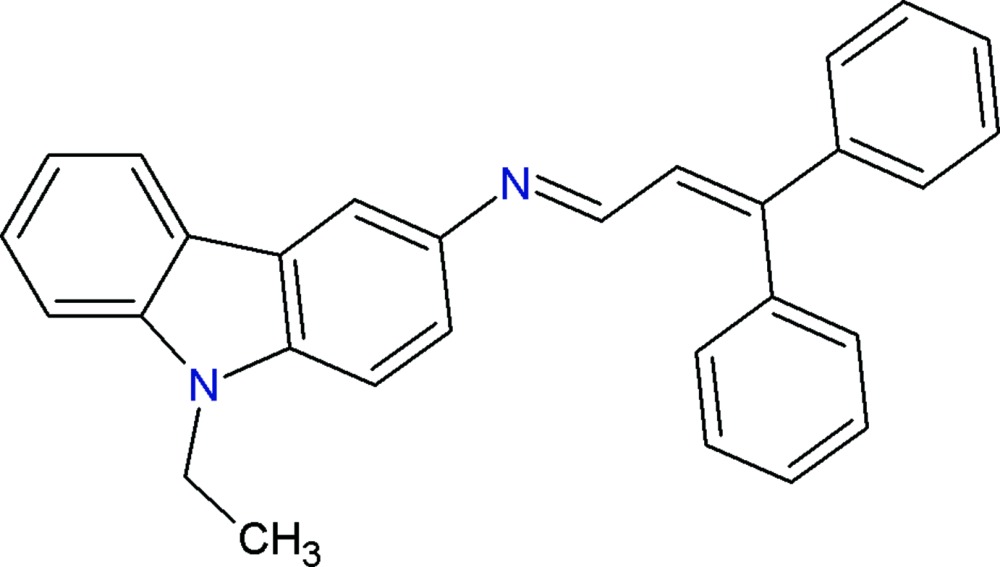


## Structural commentary   

**Figure 1:**
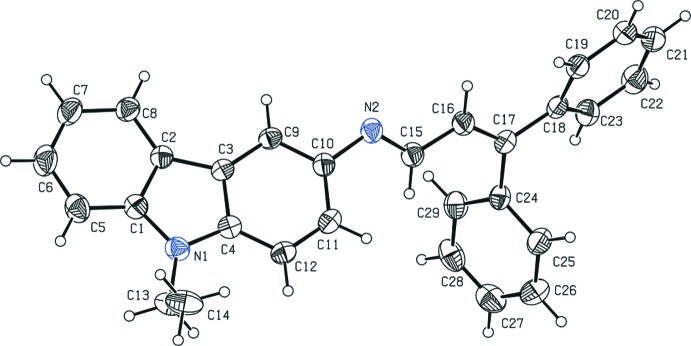
Mol­ecular structure of the title compound with the atom labelling. Displacement ellipsoids are drawn at the 30% probability level.

The mol­ecular structure of the title compound is illustrated in Fig. 1 ▸. The C15=N2 bond of the central imine group adopts an *E* conformation. The carbazole ring system (N1/C1–C12) is essentially planar [maximum deviation = 0.039 (2) Å for atom C9]. The phenyl rings C18–C23 and C24–C29 of the (3,3-di­phenyl­allyl­idene) unit are oriented at dihedral angles of 75.9 (1) and 64.6 (1)°, respectively, to the mean plane of the carbazole ring system. The dihedral angle between the two phenyl rings is 76.1 (1)°. The sum of the bond angles around atom N1 (359.7°) of the pyrrole ring is in accordance with *sp* ^2^ hybridization. The geometric parameters of the title mol­ecule agree well with those reported for similar structures (Murugavel *et al.*, 2009 ▸; Archana *et al.*, 2011 ▸).

## Supra­molecular features   

**Figure 2:**
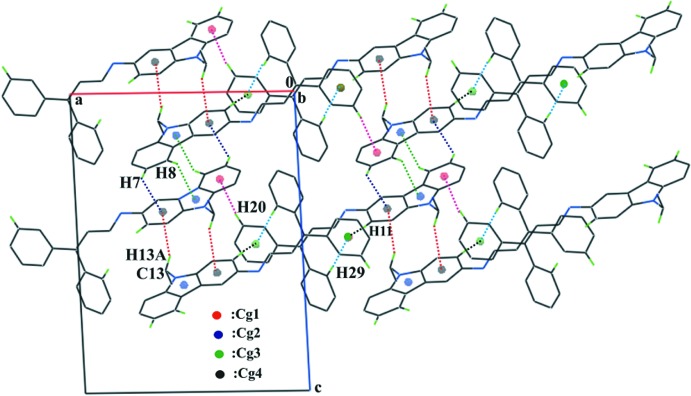
A partial view along the *b* axis of the crystal packing of the title compound, showing the inter­molecular C—H⋯π inter­actions (see Table 1 ▸ for details), forming a three-dimensional supra­molecular network. H atoms not involved in these inter­actions have been omitted for clarity.

In the crystal, mol­ecules are linked by six inter­molecular C—H⋯π inter­actions, forming a three-dimensional supra­molecular network (Table 1 ▸ and Fig. 2 ▸). Four of these inter­actions involves a benzene H atom of the carbazole ring system and a benzene ring of an adjacent mol­ecule, *viz.* C7—H7⋯*Cg*1^i^, C11—H11⋯*Cg*3^ii^, C20—H20⋯*Cg*4^iv^, and C29—H29⋯*Cg*3^v^. The other two involve a benzene H atom of the carbazole ring system and the pyrrole ring of an adjacent mol­ecule (C8—H8⋯*Cg*2^i^), and a methyl­ene H atom of the ethyl group and a benzene ring of an adjacent mol­ecule (C13—H13*A*⋯*Cg*1^iii^); see Table 1 ▸ and Fig. 2 ▸ for full details.

## Synthesis and crystallization   

A 25 ml round-bottom flask was charged with 9-ethyl-9*H*-carbazol-3-amine (1 mmol), 3,3-di­phenyl­acryl­aldehyde (1 mmol) and sulfated SnO_2_-Bi_2_O_3_-fly ash catalyst (20 mg) in water (15 ml) and the mixture was refluxed at 363 K for 1h. On completion of the reaction (monitored by TLC with ethyl acetate and hexane as an eluent 20%) the mixture was cooled to ambient temperature. Di­chloro­methane (20 ml) was then added to separate the organic and aqueous layers. The organic layer was filtered, dried on anhydrous Na_2_SO_4_ and the solvent removed using a rotary evaporator. The crude product obtained was purified by column chromatography on silica gel (200 mesh) with hexane and ethyl acetate (4:1) as eluent, to afford the title compound in good yield (93%). Red crystals suitable for X-ray diffraction analysis were obtained after recrystallization in CH_2_Cl_2_.

## Refinement   

Crystal data, data collection and structure refinement details are summarized in Table 2 ▸. H atoms were positioned geometrically and constrained to ride on their parent atom with C—H = 0.93–0.97 Å and with *U*_iso_(H) = 1.5*U*_eq_ for methyl H atoms and 1.2*U*_eq_(C) for other H atoms.

## Supplementary Material

Crystal structure: contains datablock(s) global, I. DOI: 10.1107/S2056989015005770/su5095sup1.cif

Structure factors: contains datablock(s) I. DOI: 10.1107/S2056989015005770/su5095Isup2.hkl

CCDC reference: 967497

Additional supporting information:  crystallographic information; 3D view; checkCIF report

## Figures and Tables

**Table 1:** Hydrogen-bond geometry (, ) *Cg*1, *Cg*2, *Cg*3 and *Cg*4 are the centroids of rings C3/C4/C9C12, N1/C1C4, C18C23 and C1/C2/C5C8, respectively.

*D*H*A*	*D*H	H*A*	*D* *A*	*D*H*A*
C7H7*Cg*1^i^	0.93	2.92	3.647(2)	136
C8H8*Cg*2^i^	0.93	2.98	3.777(2)	145
C11H11*Cg*3^ii^	0.93	2.85	3.551(2)	133
C13H13*A* *Cg*1^iii^	0.97	3.00	3.749(2)	135
C20H20*Cg*4^iv^	0.93	2.62	3.498(2)	157
C29H29*Cg*3^v^	0.93	2.87	3.796(3)	175

Symmetry codes: (i) 
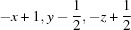
; (ii) 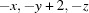; (iii) 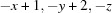; (iv) 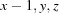; (v) 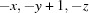.

**Table 2:** Experimental details

Crystal data	
Chemical formula	C_29_H_24_N_2_
*M*_r_	400.50
Crystal system, space group	Monoclinic, *P*2_1_/*c*
Temperature (K)	293
*a*, *b*, *c* ()	13.6502(17), 8.7616(13), 18.224(2)
()	92.234(11)
*V* (^3^)	2177.9(5)
*Z*	4
Radiation type	Mo *K*
(mm^1^)	0.07
Crystal size (mm)	0.23 0.21 0.15

Data collection	
Diffractometer	Bruker SMART CCD area detector
Absorption correction	Multi-scan (*SADABS*; Sheldrick, 1996 ▸)
*T*_min_, *T*_max_	0.984, 0.989
No. of measured, independent and observed [*I* > 2(*I*)] reflections	9709, 4982, 3066
*R*_int_	0.047
(sin /)_max_ (^1^)	0.688

Refinement	
*R*[*F* ^2^ > 2(*F* ^2^)], *wR*(*F* ^2^), *S*	0.056, 0.150, 1.03
No. of reflections	4982
No. of parameters	280
H-atom treatment	H-atom parameters constrained
_max_, _min_ (e ^3^)	0.17, 0.19

Computer programs: *SMART* and *SAINT* (Bruker, 2002 ▸), *SHELXS97* and *SHELXL97* (Sheldrick, 2008 ▸), *ORTEP-3 for Windows* (Farrugia (2012 ▸) and *PLATON* (Spek, 2009 ▸).

## supplementary crystallographic information

### Crystal data

**Table:**

C_29_H_24_N_2_	*F*(000) = 848
*M**_r_* = 400.50	*D*_x_ = 1.221 Mg m^−^^3^
Monoclinic, *P*2_1_/*c*	Mo *K*α radiation, λ = 0.71073 Å
Hall symbol: -P 2ybc	Cell parameters from 5927 reflections
*a* = 13.6502 (17) Å	θ = 2.8–29.3°
*b* = 8.7616 (13) Å	µ = 0.07 mm^−^^1^
*c* = 18.224 (2) Å	*T* = 293 K
β = 92.234 (11)°	Block, red
*V* = 2177.9 (5) Å^3^	0.23 × 0.21 × 0.15 mm
*Z* = 4	

### Data collection

**Table:**

Bruker SMART CCD area-detector diffractometer	4982 independent reflections
Radiation source: fine-focus sealed tube	3066 reflections with *I* > 2σ(*I*)
Graphite monochromator	*R*_int_ = 0.047
φ and ω scans	θ_max_ = 29.3°, θ_min_ = 2.8°
Absorption correction: multi-scan (*SADABS*; Sheldrick, 1996)	*h* = −15→18
*T*_min_ = 0.984, *T*_max_ = 0.989	*k* = −11→11
9709 measured reflections	*l* = −24→23

### Refinement

**Table:**

Refinement on *F*^2^	Primary atom site location: structure-invariant direct methods
Least-squares matrix: full	Secondary atom site location: difference Fourier map
*R*[*F*^2^ > 2σ(*F*^2^)] = 0.056	Hydrogen site location: inferred from neighbouring sites
*wR*(*F*^2^) = 0.150	H-atom parameters constrained
*S* = 1.03	*w* = 1/[σ^2^(*F*_o_^2^) + (0.0555*P*)^2^ + 0.1709*P*] where *P* = (*F*_o_^2^ + 2*F*_c_^2^)/3
4982 reflections	(Δ/σ)_max_ < 0.001
280 parameters	Δρ_max_ = 0.17 e Å^−^^3^
0 restraints	Δρ_min_ = −0.19 e Å^−^^3^

### Special details

**Table:**

Geometry. All esds (except the esd in the dihedral angle between two l.s. planes) are estimated using the full covariance matrix. The cell esds are taken into account individually in the estimation of esds in distances, angles and torsion angles; correlations between esds in cell parameters are only used when they are defined by crystal symmetry. An approximate (isotropic) treatment of cell esds is used for estimating esds involving l.s. planes.
Refinement. Refinement of F^2^ against ALL reflections. The weighted R-factor wR and goodness of fit S are based on F^2^, conventional R-factors R are based on F, with F set to zero for negative F^2^. The threshold expression of F^2^ > 2sigma(F^2^) is used only for calculating R-factors(gt) etc. and is not relevant to the choice of reflections for refinement. R-factors based on F^2^ are statistically about twice as large as those based on F, and R- factors based on ALL data will be even larger.

### Fractional atomic coordinates and isotropic or equivalent isotropic displacement parameters (Å^2^)

**Table:**

	*x*	*y*	*z*	*U*_iso_*/*U*_eq_	
C14	0.5899 (2)	1.3222 (3)	0.14013 (13)	0.0822 (7)	
H14A	0.6192	1.4132	0.1215	0.123*	
H14B	0.6144	1.3041	0.1894	0.123*	
H14C	0.5200	1.3346	0.1399	0.123*	
N2	0.19996 (10)	0.8129 (2)	0.10030 (8)	0.0516 (4)	
C10	0.29431 (12)	0.8778 (2)	0.09957 (9)	0.0454 (4)	
C9	0.36711 (12)	0.8093 (2)	0.14234 (9)	0.0449 (4)	
H9	0.3538	0.7226	0.1696	0.054*	
C4	0.47794 (13)	1.0054 (2)	0.10553 (9)	0.0462 (4)	
C3	0.46026 (12)	0.8713 (2)	0.14420 (9)	0.0427 (4)	
N1	0.57338 (11)	1.04985 (18)	0.11800 (8)	0.0530 (4)	
C12	0.40455 (14)	1.0746 (2)	0.06391 (10)	0.0530 (5)	
H12	0.4166	1.1636	0.0380	0.064*	
C11	0.31382 (14)	1.0100 (2)	0.06153 (10)	0.0518 (5)	
H11	0.2636	1.0560	0.0336	0.062*	
C19	−0.16510 (13)	0.7140 (2)	0.04772 (10)	0.0533 (5)	
H19	−0.1369	0.7691	0.0867	0.064*	
C15	0.14631 (13)	0.8153 (2)	0.04164 (10)	0.0529 (5)	
H15	0.1714	0.8548	−0.0011	0.063*	
C2	0.55154 (13)	0.8316 (2)	0.18155 (9)	0.0455 (4)	
C18	−0.11167 (13)	0.6882 (2)	−0.01430 (10)	0.0471 (4)	
C1	0.61869 (13)	0.9460 (2)	0.16451 (10)	0.0510 (5)	
C16	0.04842 (13)	0.7586 (3)	0.04026 (10)	0.0553 (5)	
H16	0.0230	0.7319	0.0851	0.066*	
C23	−0.15719 (14)	0.6089 (2)	−0.07131 (11)	0.0588 (5)	
H23	−0.1235	0.5911	−0.1139	0.071*	
C20	−0.25842 (14)	0.6598 (3)	0.05254 (12)	0.0614 (6)	
H20	−0.2930	0.6772	0.0947	0.074*	
C24	0.02709 (12)	0.7604 (2)	−0.09425 (9)	0.0477 (5)	
C13	0.61468 (15)	1.1902 (2)	0.09293 (11)	0.0614 (5)	
H13A	0.5906	1.2099	0.0430	0.074*	
H13B	0.6854	1.1800	0.0923	0.074*	
C8	0.58185 (14)	0.7119 (2)	0.22603 (10)	0.0550 (5)	
H8	0.5385	0.6343	0.2374	0.066*	
C29	0.10181 (14)	0.6715 (3)	−0.11811 (11)	0.0592 (5)	
H29	0.1314	0.6011	−0.0862	0.071*	
C28	0.13409 (15)	0.6844 (3)	−0.18854 (13)	0.0714 (7)	
H28	0.1846	0.6220	−0.2037	0.086*	
C5	0.71384 (14)	0.9426 (3)	0.19254 (11)	0.0666 (6)	
H5	0.7580	1.0194	0.1817	0.080*	
C22	−0.25053 (16)	0.5561 (3)	−0.06653 (13)	0.0688 (6)	
H22	−0.2800	0.5034	−0.1058	0.083*	
C25	−0.01406 (15)	0.8636 (3)	−0.14288 (11)	0.0639 (6)	
H25	−0.0655	0.9249	−0.1284	0.077*	
C21	−0.30082 (16)	0.5802 (3)	−0.00460 (13)	0.0675 (6)	
H21	−0.3641	0.5423	−0.0012	0.081*	
C27	0.09292 (18)	0.7869 (3)	−0.23570 (12)	0.0767 (7)	
H27	0.1145	0.7952	−0.2833	0.092*	
C6	0.74105 (16)	0.8237 (3)	0.23656 (12)	0.0746 (7)	
H6	0.8050	0.8196	0.2560	0.089*	
C26	0.01946 (18)	0.8779 (3)	−0.21270 (12)	0.0784 (7)	
H26	−0.0083	0.9503	−0.2445	0.094*	
C7	0.67657 (16)	0.7091 (3)	0.25318 (11)	0.0699 (6)	
H7	0.6977	0.6289	0.2832	0.084*	
C17	−0.00988 (13)	0.7403 (2)	−0.01930 (9)	0.0478 (4)	

### Atomic displacement parameters (Å^2^)

**Table:**

	*U*^11^	*U*^22^	*U*^33^	*U*^12^	*U*^13^	*U*^23^
C14	0.116 (2)	0.0536 (15)	0.0788 (15)	−0.0230 (14)	0.0288 (14)	−0.0040 (12)
N2	0.0456 (9)	0.0658 (12)	0.0433 (8)	0.0018 (8)	−0.0019 (7)	−0.0031 (8)
C10	0.0456 (10)	0.0533 (12)	0.0372 (9)	0.0019 (8)	0.0004 (7)	−0.0053 (8)
C9	0.0533 (11)	0.0435 (11)	0.0377 (9)	−0.0022 (8)	−0.0017 (8)	−0.0005 (8)
C4	0.0522 (11)	0.0419 (11)	0.0444 (9)	0.0002 (8)	0.0019 (8)	−0.0038 (8)
C3	0.0499 (10)	0.0401 (10)	0.0380 (9)	0.0004 (8)	−0.0001 (7)	−0.0022 (8)
N1	0.0532 (9)	0.0478 (10)	0.0578 (9)	−0.0083 (8)	0.0017 (7)	−0.0004 (8)
C12	0.0614 (12)	0.0441 (12)	0.0535 (11)	0.0019 (9)	0.0023 (9)	0.0054 (9)
C11	0.0568 (11)	0.0526 (12)	0.0455 (10)	0.0101 (9)	−0.0034 (8)	0.0013 (9)
C19	0.0526 (11)	0.0567 (13)	0.0504 (10)	0.0044 (9)	−0.0021 (8)	0.0045 (9)
C15	0.0484 (10)	0.0683 (14)	0.0418 (10)	0.0015 (9)	−0.0006 (8)	−0.0023 (9)
C2	0.0482 (10)	0.0488 (11)	0.0393 (9)	0.0001 (8)	0.0002 (7)	−0.0041 (8)
C18	0.0471 (10)	0.0461 (11)	0.0474 (10)	0.0048 (8)	−0.0057 (8)	0.0054 (8)
C1	0.0532 (11)	0.0524 (12)	0.0472 (10)	−0.0032 (9)	−0.0009 (8)	−0.0064 (9)
C16	0.0472 (10)	0.0735 (15)	0.0451 (10)	0.0008 (10)	−0.0007 (8)	0.0036 (10)
C23	0.0559 (12)	0.0603 (14)	0.0596 (12)	0.0007 (10)	−0.0046 (9)	−0.0024 (10)
C20	0.0531 (12)	0.0642 (15)	0.0671 (13)	0.0050 (10)	0.0057 (10)	0.0154 (11)
C24	0.0446 (10)	0.0522 (12)	0.0459 (10)	0.0039 (9)	−0.0039 (8)	−0.0050 (9)
C13	0.0704 (13)	0.0566 (14)	0.0584 (12)	−0.0141 (11)	0.0164 (10)	0.0004 (10)
C8	0.0571 (11)	0.0621 (14)	0.0456 (10)	0.0048 (10)	0.0005 (8)	0.0032 (9)
C29	0.0545 (12)	0.0603 (14)	0.0626 (12)	0.0056 (10)	0.0008 (9)	−0.0078 (10)
C28	0.0530 (12)	0.0880 (19)	0.0741 (15)	−0.0027 (12)	0.0132 (11)	−0.0268 (14)
C5	0.0539 (12)	0.0804 (17)	0.0649 (13)	−0.0121 (11)	−0.0057 (10)	−0.0085 (12)
C22	0.0697 (14)	0.0616 (15)	0.0738 (14)	−0.0076 (11)	−0.0155 (12)	−0.0035 (12)
C25	0.0635 (13)	0.0712 (15)	0.0568 (12)	0.0143 (11)	−0.0012 (10)	0.0049 (11)
C21	0.0553 (12)	0.0585 (15)	0.0879 (16)	−0.0069 (10)	−0.0070 (12)	0.0194 (13)
C27	0.0734 (15)	0.108 (2)	0.0490 (12)	−0.0204 (15)	0.0074 (11)	−0.0124 (13)
C6	0.0600 (13)	0.101 (2)	0.0610 (13)	0.0021 (13)	−0.0172 (11)	−0.0034 (14)
C26	0.0879 (17)	0.092 (2)	0.0542 (13)	0.0018 (15)	−0.0057 (12)	0.0154 (13)
C7	0.0667 (14)	0.0886 (18)	0.0534 (12)	0.0103 (13)	−0.0094 (10)	0.0095 (12)
C17	0.0488 (10)	0.0488 (12)	0.0454 (10)	0.0066 (9)	−0.0037 (8)	0.0012 (9)

### Geometric parameters (Å, º)

**Table:**

C14—C13	1.488 (3)	C18—C23	1.378 (3)
N2—C15	1.272 (2)	C18—C17	1.469 (2)
N2—C10	1.408 (2)	C1—C5	1.377 (3)
C10—C9	1.376 (2)	C16—C17	1.330 (2)
C10—C11	1.381 (3)	C23—C22	1.361 (3)
C9—C3	1.382 (2)	C20—C21	1.364 (3)
C4—N1	1.370 (2)	C24—C29	1.367 (3)
C4—C12	1.374 (2)	C24—C25	1.371 (3)
C4—C3	1.396 (3)	C24—C17	1.485 (2)
C3—C2	1.439 (2)	C8—C7	1.367 (3)
N1—C1	1.374 (2)	C29—C28	1.378 (3)
N1—C13	1.435 (2)	C28—C27	1.351 (3)
C12—C11	1.361 (3)	C5—C6	1.358 (3)
C19—C20	1.365 (3)	C22—C21	1.360 (3)
C19—C18	1.387 (3)	C25—C26	1.375 (3)
C15—C16	1.425 (2)	C27—C26	1.360 (3)
C2—C8	1.379 (3)	C6—C7	1.377 (3)
C2—C1	1.401 (3)		
			
C15—N2—C10	118.81 (16)	N1—C1—C5	129.66 (19)
C9—C10—C11	120.03 (17)	N1—C1—C2	109.14 (15)
C9—C10—N2	117.33 (17)	C5—C1—C2	121.20 (19)
C11—C10—N2	122.53 (16)	C17—C16—C15	125.96 (18)
C10—C9—C3	119.10 (17)	C22—C23—C18	121.2 (2)
N1—C4—C12	129.36 (18)	C21—C20—C19	119.9 (2)
N1—C4—C3	109.73 (15)	C29—C24—C25	117.50 (18)
C12—C4—C3	120.90 (17)	C29—C24—C17	120.63 (17)
C9—C3—C4	119.69 (16)	C25—C24—C17	121.82 (17)
C9—C3—C2	134.08 (17)	N1—C13—C14	112.39 (17)
C4—C3—C2	106.19 (16)	C7—C8—C2	119.0 (2)
C4—N1—C1	108.43 (15)	C24—C29—C28	121.3 (2)
C4—N1—C13	125.00 (17)	C27—C28—C29	120.4 (2)
C1—N1—C13	126.23 (16)	C6—C5—C1	117.9 (2)
C11—C12—C4	118.49 (18)	C21—C22—C23	120.3 (2)
C12—C11—C10	121.74 (17)	C24—C25—C26	121.0 (2)
C20—C19—C18	121.14 (19)	C22—C21—C20	120.0 (2)
N2—C15—C16	121.17 (18)	C28—C27—C26	119.2 (2)
C8—C2—C1	119.35 (17)	C5—C6—C7	121.8 (2)
C8—C2—C3	134.14 (18)	C27—C26—C25	120.5 (2)
C1—C2—C3	106.50 (16)	C8—C7—C6	120.7 (2)
C23—C18—C19	117.43 (18)	C16—C17—C18	121.61 (17)
C23—C18—C17	120.66 (17)	C16—C17—C24	121.49 (17)
C19—C18—C17	121.91 (16)	C18—C17—C24	116.75 (15)
			
C15—N2—C10—C9	−145.48 (18)	N2—C15—C16—C17	−172.1 (2)
C15—N2—C10—C11	38.4 (3)	C19—C18—C23—C22	−0.9 (3)
C11—C10—C9—C3	−2.6 (3)	C17—C18—C23—C22	178.05 (19)
N2—C10—C9—C3	−178.80 (15)	C18—C19—C20—C21	−0.7 (3)
C10—C9—C3—C4	2.9 (2)	C4—N1—C13—C14	79.5 (2)
C10—C9—C3—C2	179.98 (18)	C1—N1—C13—C14	−93.1 (2)
N1—C4—C3—C9	177.03 (15)	C1—C2—C8—C7	0.8 (3)
C12—C4—C3—C9	−1.8 (3)	C3—C2—C8—C7	180.0 (2)
N1—C4—C3—C2	−0.8 (2)	C25—C24—C29—C28	−0.5 (3)
C12—C4—C3—C2	−179.66 (16)	C17—C24—C29—C28	177.20 (19)
C12—C4—N1—C1	178.78 (19)	C24—C29—C28—C27	0.7 (3)
C3—C4—N1—C1	0.1 (2)	N1—C1—C5—C6	−178.4 (2)
C12—C4—N1—C13	5.1 (3)	C2—C1—C5—C6	0.8 (3)
C3—C4—N1—C13	−173.63 (17)	C18—C23—C22—C21	−0.4 (3)
N1—C4—C12—C11	−178.20 (17)	C29—C24—C25—C26	−0.6 (3)
C3—C4—C12—C11	0.4 (3)	C17—C24—C25—C26	−178.3 (2)
C4—C12—C11—C10	−0.1 (3)	C23—C22—C21—C20	1.2 (3)
C9—C10—C11—C12	1.2 (3)	C19—C20—C21—C22	−0.6 (3)
N2—C10—C11—C12	177.20 (17)	C29—C28—C27—C26	0.3 (3)
C10—N2—C15—C16	−176.66 (18)	C1—C5—C6—C7	0.0 (3)
C9—C3—C2—C8	4.6 (3)	C28—C27—C26—C25	−1.4 (4)
C4—C3—C2—C8	−178.0 (2)	C24—C25—C26—C27	1.6 (4)
C9—C3—C2—C1	−176.15 (19)	C2—C8—C7—C6	0.0 (3)
C4—C3—C2—C1	1.23 (19)	C5—C6—C7—C8	−0.5 (4)
C20—C19—C18—C23	1.4 (3)	C15—C16—C17—C18	−176.78 (19)
C20—C19—C18—C17	−177.52 (18)	C15—C16—C17—C24	7.9 (3)
C4—N1—C1—C5	−179.9 (2)	C23—C18—C17—C16	−152.0 (2)
C13—N1—C1—C5	−6.3 (3)	C19—C18—C17—C16	26.9 (3)
C4—N1—C1—C2	0.8 (2)	C23—C18—C17—C24	23.6 (3)
C13—N1—C1—C2	174.34 (17)	C19—C18—C17—C24	−157.54 (18)
C8—C2—C1—N1	178.13 (16)	C29—C24—C17—C16	59.9 (3)
C3—C2—C1—N1	−1.2 (2)	C25—C24—C17—C16	−122.5 (2)
C8—C2—C1—C5	−1.3 (3)	C29—C24—C17—C18	−115.7 (2)
C3—C2—C1—C5	179.36 (17)	C25—C24—C17—C18	61.9 (3)

### Hydrogen-bond geometry (Å, º)

*Cg*1, *Cg*2, *Cg*3 and *Cg*4 are the centroids of rings C3/C4/C9–C12, N1/C1–C4, C18–C23 and C1/C2/C5–C8, respectively.

**Table:**

*D*—H···*A*	*D*—H	H···*A*	*D*···*A*	*D*—H···*A*
C7—H7···*Cg*1^i^	0.93	2.92	3.647 (2)	136
C8—H8···*Cg*2^i^	0.93	2.98	3.777 (2)	145
C11—H11···*Cg*3^ii^	0.93	2.85	3.551 (2)	133
C13—H13*A*···*Cg*1^iii^	0.97	3.00	3.749 (2)	135
C20—H20···*Cg*4^iv^	0.93	2.62	3.498 (2)	157
C29—H29···*Cg*3^v^	0.93	2.87	3.796 (3)	175

Symmetry codes: (i) −*x*+1, *y*−1/2, −*z*+1/2; (ii) −*x*, −*y*+2, −*z*; (iii) −*x*+1, −*y*+2, −*z*; (iv) *x*−1, *y*, *z*; (v) −*x*, −*y*+1, −*z*.
